# Combination Therapy With Zoledronic Acid and FES‐Row Training Mitigates Bone Loss in Paralyzed Legs: Results of a Randomized Comparative Clinical Trial

**DOI:** 10.1002/jbm4.10167

**Published:** 2019-02-20

**Authors:** L R Morse, K L Troy, Y Fang, N Nguyen, R Battaglino, R F Goldstein, R Gupta, J A Taylor

**Affiliations:** ^1^ Rocky Mountain Regional Spinal Injury System Craig Rehabilitation Hospital Englewood CO USA; ^2^ Department of PMR University of Colorado School of Medicine Aurora CO USA; ^3^ Department of Biomedical Engineering Worcester Polytechnic Institute Worcester MA USA; ^4^ Spaulding Rehabilitation Hospital Boston MA USA; ^5^ Department of Physical Medicine and Rehabilitation Harvard Medical School Boston MA USA; ^6^ Department of Radiology Massachusetts General Hospital Boston MA USA

**Keywords:** OSTEOPOROSIS, THERAPEUTICS, ANTIRESORPTIVES, CLINICAL TRIALS, BONE QCT

## Abstract

Spinal cord injury (SCI) results in rapid, severe osteoporosis and an increased risk of lower extremity fractures. Despite the medical complications associated with these fractures, there is no standard of care to prevent osteoporotic fractures following SCI. Functional electrical stimulation‐ (FES‐) assisted rowing is a promising intervention to improve bone health in SCI because of its ability to generate a muscular contraction in conjunction with mechanical loading of the lower extremity long bones. Combination therapy consisting of FES‐rowing plus zoledronic acid (ZA) may be a superior treatment via inhibition of bone resorption and stimulation of new bone formation. We studied participants enrolled in a randomized clinical trial comparing FES‐rowing alone with FES‐rowing plus ZA to improve bone health in SCI. Volumetric CT scans at the distal femur and proximal tibial metaphyses were performed. Bone geometric properties (cortical thickness index [CTI], cortical compressive strength index [CSI], buckling ratio [BR], bending strength index) and mineral (cortical bone volume [CBV], cortical bone mineral density, cortical bone mineral content) indices were determined. In models adjusting for baseline values, we found that the CBV (*p *= 0.05 to 0.006), the CTI (*p* = 0.009), and the BR (*p* = 0.001) at both the distal femoral and proximal tibial metaphyses were greater in the ZA plus rowing group compared with the rowing‐only group. Similarly, there was a significant positive association between the total rowing work completed and the BR at the proximal tibia (*p* = 0.05). A subgroup analysis of the rowing‐only arm showed that gains in the CSI at the tibial metaphysis varied in a dose‐dependent fashion based on the total amount of exercise performed (*p* = 0.009). These findings demonstrate that the osteogenic response to FES‐rowing is dose‐dependent. Combination therapy with ZA and FES‐row training has therapeutic potential to improve bone quality, and perhaps reduce fracture risk at the most common fracture site following SCI. © 2019 The Authors. *JBMR Plus* Published by Wiley Periodicals, Inc. on behalf of the American Society for Bone and Mineral Research.

## Introduction

Osteoporosis following spinal cord injury (SCI) is thought to be based primarily on the loss of mechanical loading that occurs after lower extremity paralysis.^1^, ^2^, ^3^ Bone loss leads to fractures in up to 50% of individuals with SCI, with the majority of fractures occurring at the metaphyses of the proximal tibia and distal femur. Fractures are associated with prolonged immobility; worsening disability; serious medical complications, including pressure ulcer formation and amputation^4^; and increased mortality.^5^ Despite the clinical significance of disuse osteoporosis, treatment options are limited, and there is controversy in the field regarding the efficacy of antiresorptive agents after SCI.^6^

Despite the lack of consensus regarding antiresorptive medications in SCI, bisphosphonates are the class of medication most commonly prescribed to treat SCI‐induced osteoporosis.^7^ Although there is evidence suggesting that these drugs mitigate bone loss after SCI,^8^, ^9^, ^10^, ^11^ they do not stimulate new bone formation. To date, there have been few therapeutic interventions shown to stimulate new bone formation, increase bone mass, improve bone microarchitecture, or improve bone strength after SCI.^12^, ^13^, ^14^ Bone is a dynamic organ that modulates the rate of new bone formation in response to varying levels of mechanical strain. Weight‐bearing exercises have been shown to increase bone density, cortical thickness, and bone strength in the general population.^15^, ^16^, ^17^, ^18^, ^19^ Because lower extremity paralysis is a contributing factor to disuse osteoporosis, reintroduction of mechanical loading may effectively stimulate bone regeneration.

For individuals with SCI, functional electrical stimulation‐ (FES‐) rowing exercise may provide sufficient mechanical loading of the paralyzed lower limbs to stimulate bone formation. FES‐row training entails stimulation of the hamstrings and quadriceps with electricity in a cyclical fashion so that the paralyzed legs flex and extend to assist the intact arms during a rowing cycle. Several animal and human studies have shown new bone formation based on FES alone.^20^, ^21^ Slowing of bone loss has been reported in response to FES‐cycling.^22^ The primary aim of this study was to test the osteoanabolic effects of a novel therapeutic exercise, either alone or in combination with an antiresorptive medication. We therefore conducted a clinical trial testing combination therapy with FES‐rowing plus a bisphosphonate (zoledronic acid [ZA]) compared with FES‐rowing alone to improve bone in the paralyzed lower extremity. We hypothesized that a combination therapy would result in greater gains in bone than FES‐rowing alone. We also hypothesized that gains with rowing would be dose‐dependent, in that greater rowing work would yield greater gains in bone.

## Participants and Methods

### Participants and clinical trial design

We conducted a comparative clinical trial where nonambulatory men and women with SCI were randomized by simple randomization to one of the following arms: (1) a 12‐month FES‐rowing‐exercise program (rowing alone), or (2) a combination treatment consisting of a 12‐month FES‐rowing‐exercise program plus a 1‐time dose of zoledronic acid (FES‐rowing + ZA). This trial was registered at http://clinicaltrials.gov/show/NCT01426555 (FES‐Rowing Versus Zoledronic Acid to Improve Bone Health in Spinal Cord Injury [SCI]). Volunteers were individuals who had received care at our outpatient rehabilitation center or our Veterans Affairs (VA) medical center. All volunteers were 18 years or older, had an American Spinal Injury Association Impairment Scale (AIS) A, B, or C SCI at cervical level 4 or lower, were injured for 18 months or more, and used a wheelchair as the primary mobility mode. Volunteers were ineligible for the study if they were actively being treated for epilepsy; actively using medications potentially affecting bone metabolism, including parathyroid hormone (PTH) and PTH analogs, bisphosphonates, androgenic steroids, estrogenic steroids, antiepileptics, lithium, or oral glucocorticoid (use for more than 3 months); if they had a history of peripheral nerve compression or rotator cuff injury that limited the ability to exercise; uncontrolled diabetes; active renal disease; implanted defibrillator or pacemaker; an active grade 2 or greater pressure ulcer in a location that could be worsened with exercise; or if they had an active bone fracture or lower extremity contractures. Because osteonecrosis of the jaw is a rare complication of bisphosphonate use, participants were excluded if they had planned invasive dental procedures such as tooth extractions or dental implants (excluding routine cleaning). Those who reported bisphosphonate use within the year prior to enrollment were also excluded. Our institutional review boards approved all protocols, and all participants gave their written informed consent to participate. All adverse events were reviewed by the study's medical monitor to determine study relatedness.

### Primary and secondary outcomes

The primary outcomes were change in bone mass and bone geometry at the distal femur and proximal tibia as measured by volumetric quantitative computed tomography (vQCT) at baseline and after 12 months of FES‐rowing with or without ZA. The secondary outcome was change in bone density at the distal femur and proximal tibia as measured by DXA at baseline and after 12 months of FES‐rowing with or without ZA. Prior to completion of the trial the center collecting DXA data withdrew as a study site. Therefore, the secondary outcome could not be assessed in this study.

### Sample size calculations

Sample size calculations were based on the anticipated effects of FES‐rowing and the effects of FES‐rowing plus ZA on cortical thickness measured by vQCT. Based on pilot data collected for 2 participants prior to initiation of the trial, we calculated the difference in cortical thickness measured by vQCT at the proximal tibia before and after rowing for 6 months. In 1 subject, the average change in cortical thickness was 0.6936 cm (SD = 0.0953) and in the other subject, the average change in cortical thickness was 0.7369 cm (SD = 0.1164). Based on these findings, 6 participants per arm were needed to detect a change of approximately 20% of this difference over a 12‐month period (0.14 cm) and assuming a SD of 0.10 cm (similar to the SD in the difference of cortical thickness in these 2 subjects), at a power of 90% and α = 0.05. Furthermore, we expected to detect as little as a 0.18‐cm difference in cortical thickness between each treatment group (ZA + rowing vs. rowing alone) at a power of 90% and α = 0.05 with 8 participants in each group, assuming a SD = 0.1 cm. Recruitment goals were set substantially higher than this (36 per arm) to account for expected attrition and wide variations in the duration of row training.

### Medical clearance, secondary screening, and medical monitor

All enrolled participants underwent a physical exam by the study physician. Motor level and completeness of injury were confirmed by physical exam at study entry by the study physician according to the American Spinal Injury Association Impairment Scale (AIS). Participants were classified as AIS A (sensory and motor complete, no sensory or motor function below the neurological level of injury = 18 individuals), AIS B (motor complete, preservation of sensory but no motor function below the neurological level of injury = 6 individuals), or AIS C (motor incomplete, sensory and motor function preserved below the neurological level, and more than half the key muscles below the neurological level not strong enough to overcome gravity = 5 individuals).

Because rare cases of atrial fibrillation have been reported in women after ZA infusion, all participants underwent electrocardiogram testing to assess for this prior to infusion. The presence of atrial fibrillation was not an indication for removal from the study. Any participant age 40 years or older with a family history of high blood pressure, sudden cardiac death, heart attack, coronary bypass surgery, stroke, diabetes, or obesity, and any participant aged 55 years or older required cardiac clearance by the study cardiologist to participate in the study. Screening lab work was performed by our clinical reference laboratory. Participants were screened for renal insufficiency with glomerular filtration rate >35 mL/min as the threshold to continue in the study. Enrolled participants were also screened for calcium abnormalities and vitamin D deficiency. Those with hypercalcemia (>10.5 mg/dL) or hypocalcemia (<8.5 mg/dL) were removed from the study to avoid exacerbation with ZA administration. Those with vitamin D deficiency (25‐OH vitamin D <30 ng/mL) underwent repletion with 50,000 IU of ergocalciferol weekly for 8 weeks. Vitamin D levels were then rechecked. This was repeated until vitamin D levels were >30 ng/mL. All enrolled participants were provided with daily calcium (1500 mg/day) and vitamin D (1000 IU/day) supplementation for the duration of the study.

### Functional electrical stimulation‐row training

FES‐rowing requires adaptations to an existing rower (Concept 2, Morrisville, VT, USA) that involve a seating system that provides trunk stability and constrains leg motion to the sagittal plane. In addition, there is a button on the handle of the rower that provides a command signal to an electrical stimulator (Odstock, Salisbury, England) to initiate rowing and control the timing of leg muscle stimulation (no ramp, pulse width = 450 ms, frequency = 40 Hz). The exercising individual synchronizes upper body movement with the FES‐controlled leg movement via a voluntary thumb press to control the timing of stimulation to the paralyzed leg muscles. The specifics of this device have been described elsewhere.^23^

To perform FES‐row training, muscle strength and endurance is first developed in the paralyzed legs via an initial period of FES‐strength training for the quadriceps and hamstring muscle groups. Electrodes over the motor points of the quadriceps (rectus femoris, vastus medialis, vastus lateralis), and hamstrings (biceps femoris and semitendinosus) attached to the 4‐channel stimulator provided alternating contractions of the quadriceps and hamstrings (6 s per contraction) for full‐knee extension and hamstrings isometric contraction. Frequency of training was 3 to 5 days/week and the duration increased to the point where repetitive full‐knee extension for 30 min could be achieved. At this point, participants then began the 1‐year course of FES‐row training. Training consisted of intervals of FES‐rowing that continually increased with the goal of 30 min of continuous FES‐rowing 3 days/week at an intensity of 75% to 85% of peak heart rate. Training data were monitored on a weekly basis.

### Zoledronic acid administration

Participants randomized to the combination arm (rowing + ZA) received a 1‐time infusion of ZA (5 m/100 mg solution infused over 15 min) after strength training and when the 1‐year row‐training course was initiated. Participants developing acute‐phase‐reaction symptoms following the infusion (transient mild fever, headache, myalgias, arthralgias, or flu‐like symptoms) were encouraged to take acetaminophen or ibuprofen.

### Volumetric quantitative computed tomography analysis

The distal femur and proximal tibia were scanned twice (start of row‐training and completion of row‐training). The scans were acquired using one of two scanners: (1) a 128‐slice multidetector CT scanner (Definition Flash, Siemens Medical Systems, Forchheim, Germany; 170 to 200 mAs, 120 kVp, in‐plane pixel resolution 0.3 to 0.5 mm, slice thickness 0.5 mm), or (2) a 16‐slice CT scanner (LightSpeed Pro 16, GE Healthcare, Chicago, IL, USA; 120 kVp, 50 mAs, pixel resolution 0.652 to 0.977 mm, slice thickness 0.625 to 1.250 mm). For each participant, both baseline and end‐of‐study scans were obtained on the same machine. Each scan included a calibration phantom (MindWays, Austin, TX, USA) in the imaging field to convert CT Hounsfield units (HU) to bone‐equivalent density (ρHA, g/cm^3^). Reconstructed images included the 18‐ to 25‐cm region surrounding the knee joint. After each bone was aligned anatomically, a fixed density threshold of 0.15 g/cm^3^ was used to identify the periosteal surface of bone. In some images, manual correction was required to fill in missing low‐density surface voxels; all images were processed by a single investigator (YF). Next, the epiphysis, metaphysis, and diaphysis regions of the bilateral proximal tibias and the distal femorals were defined corresponding to 0% to 10%, 10% to 20%, and 20% to 30% of segment length, respectively.^24^ The majority of scans did not include the full diaphysis; therefore, this region was not analyzed. Within each epiphysis and metaphysis segment, integral, trabecular, and cortical regions were defined for detailed analysis. The integral region consisted of all voxels enclosed within the periosteal surface; the cortical region was all voxels with ρ_ha_ >0.33 g/cm^3^ that were located within 3.5 mm of the periosteal surface; the trabecular region was all interior voxels located more than 3.5 mm from the periosteal surface.

Within each segment and region, we calculated bone mineral content (BMC; g), volumetric bone mineral density (vBMD; g/cm^3^), and bone volume (BV; cm^3^). For each segment, we also calculated a series of measures related to strength. These included bending strength index (BSI; cm^3^), compressive strength index (CSI; g^2^/cm^4^), cortical thickness index (CTI; cm), and buckling ratio (BR; unitless). BSI and CSI were calculated based on the density distribution of bone mineral within the cross‐section using methods established by Lang and colleagues.^25^ CTI was the average cortical thickness of the segment, calculated based on an ideal cylinder. BR was the ratio of the maximum distance from the center of mass to the subperiosteal bone edge / the mean cortical thickness. This index of cortical instability was based on the principle that thin‐walled tubes become unstable when the ratio of the outer diameter to wall thickness exceeds some maximum value.^26^

A precision analysis was performed to account for errors that might result from imprecise alignment and region selection, and errors that occur based on interpolation as the scans are rotated and aligned. We also wanted to account for any error introduced using our 3D‐image‐registration algorithm, which we used to align posttest scans to pretest scans so that the identical regions could be compared. Accordingly, we did the following: 10 CT scans were randomly selected. Each scan was randomly rotated in 3D by 1 to 15 degrees. Then, each rotated scan was aligned and analyzed according to our standard protocol. This included manually aligning the first scan, then using 3D‐image registration to determine the rotations required to align subsequent scans. This precision analysis provided an RMS‐CV of 0.96% for BMC, 0.91% for vBMD, and 0.6% for BV.

Of the possible 8 image sets per participant (2 sites: tibia, femur * 2 sides: left and right * 2 time points: baseline and follow‐up), 17 image sets were technically limited based on the following issues and were not included in the final analysis: segment length too short or did not include complete anatomy (*n* = 7), metal implant or other artifact in the image (*n* = 6), visible fracture noted (*n* = 4). The final analysis dataset included matched pre/postimages from the 20 participants.

### Dual X‐ray absorptiometry for bone mineral density and body composition

We used a fifth‐generation GE Healthcare iDXA DXA scanner with enCore configuration version 12.3 to determine bone density and to assess body composition at baseline and follow‐up. Total fat mass (kg) and total lean mass (kg) were calculated by the system software from whole‐body scans. Fractures were most common at the knee (distal femur or proximal tibia) after SCI. Therefore, areal bone mineral density (aBMD; g/cm^2^) was determined at both SCI‐specific (distal femur and proximal tibia) and standard (femoral neck and total hip) skeletal sites as previously described.^10^ As a standard procedure, a phantom, supplied by the manufacturer providing three different densities (0.5, 1.0, and 1.5 g/cm^2^), was measured daily and accuracy was within 0.003 g/cm^2^. The RMS‐CV was 2.3% and the RMS‐SD was 0.012 g/cm^2^ at the distal femur. At the proximal tibia, the RMS‐CV was 2.4%, and the RMS‐SD was 0.028 g/cm^2^.

### Variable definition

Information regarding SCI, medical history, medication use, and fracture history was obtained by a questionnaire at the time of enrollment. Age and BMI were considered as continuous variables. Baseline 25‐OH vitamin D level was considered a continuous variable and categorized as sufficient (≥30 ng/mL) or deficient (<30 ng/mL). For postmenopausal women and men age 50 years or older, the *T*‐score was used to classify hip bone density (total hip and femoral neck) according to the World Health Organization definitions of normal (*T*‐score ≥‐1), osteopenia (*T*‐score <‐1 and >‐2.5), and osteoporosis (*T*‐score <‐2.5). For premenopausal women and men under the age of 50 years, the *Z*‐score was used to classify bone density at the hip as normal (*Z*‐score >‐2) or as lower than expected for age and sex (*Z*‐score <‐2). Bone geometry effect sizes are presented in the following way to improve readability: CSI was converted from g^2^/cm^4^ to mg^2^/cm^4^, cortical bone volume (CBV) was converted from cm^3^ to mm^3^, and CTI was converted from cm to mm. Similarly, BR coefficients were multiplied by 1000 to improve readability. Total rowing work was summed for all participants and presented as kilowatt‐hours (kWh).

### Statistical analysis

Linear regression with cluster‐adjusted standard errors (to adjust for the multiple measures, eg, two sides, per person^27^) was used for the analysis. Before estimating any regressions, linearity of quantitative variables was checked using LOWESS (locally weighted scatterplot smoothing) curves.^28^ Because of the sample size, only simple polynomials (eq, quadratic, cubic) were included in models when suggested by LOWESS curves. Given the low power for interaction tests, none were included in the final models. A set of candidate predictors/covariates were suggested by the subject matter experts in the group and a *p*‐value threshold of 0.05 was used to determine which covariates were included in the final models (as noted in the footnotes to Tables 3 and 4). During data cleaning, 2 individuals were found to have injury durations less than 18 months. Models are presented including all participants completing the study (*n* = 20) and excluding the following subgroups: active lipophilic statin users (because of the putative osteogenic effects of these medications, *n* = 2), women (*n* = 2), and individuals with injury duration less than 18 months (*n* = 2). End‐of‐study DXA results were only available for 7 participants. Therefore, baseline values were included in the analysis (leg lean mass), but change in bone density or body composition could not be included in the analyses. Although many tests were conducted, no adjustments for multiple testing were done because of the exploratory nature of the analyses and the small sample size. All analyses were performed using Stata, version 15 (StataCorp, College Station, TX, USA).

## Results

### Participant characteristics

**Figure 1:**
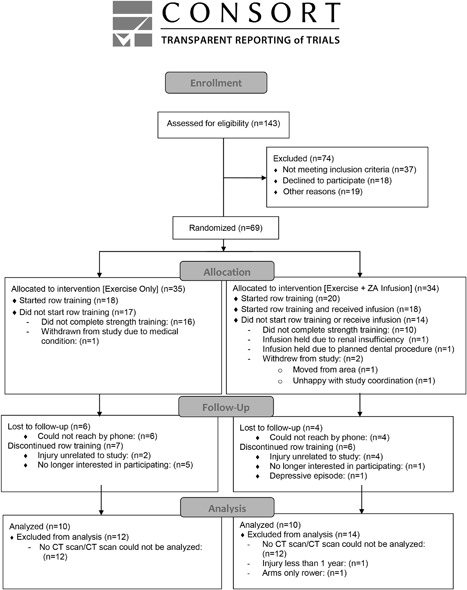
Study flow.

The trial ended when recruitment goals were met and all enrolled individuals completed participation in the study. Subject characteristics are presented in Table 1 and study flow details are presented in Figure 1. There were no baseline differences between the rowing‐only and the rowing + ZA groups. Twenty participants (2 women) completed the study between October 2010 and December 2014 and were included in the analyses. Participants were aged 38.2 ± 12.4 (SD) years (range, 22.2 to 63.5), and were 11.6 ± 12.7 years (range, 0.4 to 37.9) postinjury. Most of the participants were male (90%) and white (90%). All participants used a wheelchair as their primary mode of mobility; 75% had a motor‐complete injury. The mean BMI was 25.3 ± 5.0 kg/m^2^; 55% of participants had normal vitamin D levels (>30 ng/mL). Nine participants were found to have a vitamin D deficiency (<30 ng/mL) and were treated with supplemental vitamin D prior to progressing in the study. On average, volunteers exercised 1.6 + 0.1 times a week. However, the total amount of exercise varied substantially among volunteers, from 0.0595 to 2.39 kWh (mean, 0.921 ± 0.703). The mean work performed did not differ significantly between the two groups.

**Table 1:** Baseline Participant Characteristics

Variable	ZA + exercise arm (*n* = 10)	Exercise‐only arm (*n* = 10)	Total cohort (*n* = 20)	*p*
Demographics				
Age (years; mean ± SD)	38.3 ± 13.6	38.2 ± 11.8	38.2 ± 12.4	0.9859
Age at injury (years; mean ± SD)	29.7 ± 13.4	24.0 ± 7.4	26.9 ± 10.9	0.2576
Years since injury (mean ± SD)	8.8 ± 11.1	14.4 ± 14.1	11.6 ± 12.7	0.3400
White (*n*%)	9 (90.0%)	9 (90.0%)	18 (90.0%)	1.0
Male (*n*%)	9 (90.0%)	9 (90.0%)	18 (90.0%)	1.0
Motor‐complete injury (*n*%)	8 (80.0%)	7 (70.0%)	15 (75.0%)	1.0
25‐OH vitamin D (ng/mL; mean ± SD)	34.9 ± 8.5	31.4 ± 6.9	33.1 ± 7.7	0.3181
Deficient (≤30 ng/mL)	4 (40.0%)	5 (50.0%)	9 (45.0%)	1.0
Normal (>30 ng/mL)	6 (60.0%)	5 (50.0%)	11 (55.0%)	
Body composition	26.0 ± 5.1	24.6 ± 5.0	25.3 ± 5.0	0.5311
BMI (kg/m^2^; mean ± SD)	81.7 ± 19.2	78.2 ± 16.9	80.0 ± 17.7	0.6735
Total body weight (kg; mean ± SD)	16.1 ± 3.8	15.8 ± 3.4	16.0 ± 3.5	0.8511
Legs lean mass (kg; mean ± SD)				
Baseline aBMD (g/cm^2^; mean ± SD)				
SCI‐specific skeletal sites	0.76 ± 0.21	0.83 ± 0.36	0.80 ± 0.29	0.6194
Distal femur	0.76 ± 0.25	0.82 ± 0.34	0.79 ± 0.29	0.6702
Proximal tibia				
Traditional skeletal sites	0.82 ± 0.18	0.85 ± 0.30	0.84 ± 0.24	0.8445
Femoral neck	0.77 ± 0.17	0.82 ± 0.29	0.80 ± 0.23	0.6638
Total hip	0.97 ± 0.09	1.00 ± 0.07	0.99 ± 0.08	0.3329
Radius				
Hip bone density classification (*n*%)	3 (30.0%)	3 (30.0%)	6 (30.0%)	1.0
Normal	3 (30.0%)	3 (30.0%)	6 (30.0%)	
Osteopenia	4 (40.0%)	4 (40.0%)	8 (40.0%)	
Osteoporosis/BMD lower than expected				
Femur bone volume/ bone geometry	12.5 ± 3.9^a^	11.9 ± 3.5^b^	12.2 ± 3.7^e^	0.6526
Cortical bone volume (cm^3^)	0.3 ± 0.1^b^	0.3 ± 0.1^d^	0.3 ± 0.1^f^	0.3735
Cortical thickness index (mm)	0.09 ± 0.03^b^	0.08 ± 0.03^d^	0.09 ± 0.03^f^	0.2753
Buckling ratio				
Tibia bone volume/ bone geometry	13.2 ± 4.0^c^	13.3 ± 4.2^d^	13.3 ± 4.1^g^	0.9763
Cortical bone volume (cm^3^)	0.4 ± 0.1^d^	0.4 ± 0.1^c^	0.4 ± 0.1^g^	0.7039
Cortical thickness index (mm)	0.13 ± 0.03^d^	0.14 ± 0.05^c^	0.13 ± 0.04^g^	0.5166
Buckling ratio				
Total rowing work (kWh; mean ± SD)	1.056 ± 0.709	0.786 ± 0.707	0.921 ± 0.703	0.4049

ZA = zoledronic acid; aBMD = areal bone mineral density.

^a^ *n* = 17 observations (left and/or right).

^b^ *n* = 18 observations (left and/or right).

^c^ *n* = 15 observations (left and/or right).

^d^ *n* = 19 observations (left and/or right).

^e^ *n* = 35 observations (left and/or right).

^f^ *n* = 37 observations (left and/or right).

^g^ *n* = 34 observations (left and/or right).

### Adverse events

There were no study‐related serious adverse events. Seven participants reported eight serious adverse events that were unrelated to study participation. These included hospitalizations for infection (*n* = 7) or tachycardia (*n* = 1) and one major depressive episode. All study‐related adverse events for all enrolled participants (*n* = 69) are summarized in Table 2. Sixty‐two percent of the participants randomized to the rowing + ZA arm reported adverse events compared with 37% in the rowing‐only arm. Fifty percent (*n* = 9 of 18) of the participants who received ZA infusion reported acute‐phase reaction symptoms including fever, muscle aches, or fatigue (nonserious and expected). For all participants these symptoms resolved with acetaminophen. Musculoskeletal pain was the most commonly reported study adverse event occurring in 20% (*n* = 14 of 69 enrolled participants) of all participants. Ten percent (*n* = 7 of 69 enrolled participants) of participants reported adverse events associated with calcium/vitamin D supplementation, including nephrolithiasis (*n* = 3).

**Table 2:** Study‐Related Adverse Events

	ZA + Exercise (*n* = 34)	Exercise Only (*n * = 35)	Total cohort (*n* = 69)
All study‐related adverse events	21 (62%)	13 (37%)	34 (49%)
Infusion‐related adverse events			
Acute‐phase reaction following ZA infusion (*n*%)	9 (26%)	NA	NA
Hypophosphatemia after infusion (*n*%)	1 (3%)	NA	NA
Calcium/vitamin D supplementation‐related adverse events			
Kidney stone formation (*n*%)	2 (6%)	1 (3%)	3 (4%)
Constipation or loose stool because of calcium/vitamin D supplementation (*n*%)	0 (0%)	2 (6%)	2 (3%)
Fatigue because of calcium supplementation (*n*%)	0 (0%)	1 (3%)	1 (1%)
Took ergocalciferol daily instead of weekly (*n*%)	0 (0%)	1 (3%)	1 (1%)
Electrical stimulation‐related adverse events			
Autonomic dysreflexia during rowing (*n*%)	3 (9%)	0 (0%)	3 (4%)
Spontaneous ejaculation with electrical stimulation (*n*%)	1 (3%)	0 (0%)	1 (1%)
Exercise‐related adverse events			
Musculoskeletal pain (shoulder, elbow, wrist, neck, back, hip, knee, ankle) (*n*%)	8 (24%)	6 (17%)	14 (20%)
Dizziness because of rowing (*n*%)	2 (6%)	3 (9%)	5 (7%)
Stage 1 or 2 pressure ulcer formation because of rowing (thumb, coccyx, ischial tuberosity, leg, foot) (*n*%)	2 (6%)	4 (11%)	6 (9%)
Tachycardia or palpitations because of rowing (*n*%)	2 (6%)	1 (3%)	3 (4%)
Hypotension because of rowing (*n*%)	1 (3%)	1 (3%)	2 (3%)
Fall from rower with no injury (*n*%)	1 (3%)	0 (0%)	1 (1%)
Skin irritation because of rower straps (*n*%)	0 (0%)	1 (3%)	1 (1%)
Nausea during rowing (*n*%)	0 (0%)	1 (3%)	1 (1%)
Other/undetermined causes of study‐related adverse events			
Increased spasticity (*n*%)	3 (9%)	1 (3%)	4 (6%)
Claustrophobia with testing (*n*%)	1 (3%)	0 (0%)	1 (1%)

ZA = zoledronic acid.

### Change in bone geometry and bone density because of combination treatment (ZA + rowing)

**Figure 2:**
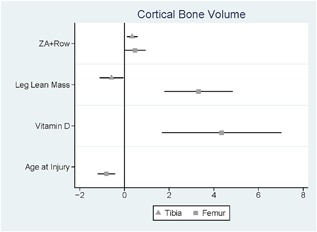
Factors associated with end‐of‐study cortical bone volume. Confidence intervals presented for models cortical bone volume (adjusted for baseline values, rowing‐only group is the reference) at the distal femur or proximal tibia. ZA = zoledronic acid.

**Figure 3:**
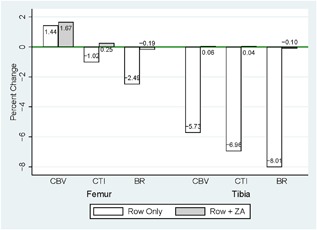
Percent change in cortical bone volume or bone geometry based on treatment group (rowing‐only or rowing + zoledronic acid). Change in cortical bone volume (CBV), cortical thickness index (CTI), and buckling ratio (BR) are presented for distal femur and proximal tibia.

In models adjusting for baseline values, we found that ZA treatment was positively associated with end‐of‐study CBV (Table 3 and Fig. 2). ZA users had 345 ± 109 mm^3^ greater CBV at the proximal tibial metaphysis (*p* = 0.006) and 471 ± 225 mm^3^ at the distal femoral metaphysis (*p* = 0.05) than those in the rowing‐only arm. This corresponded to mitigation of 5.7% loss of CBV at the proximal tibia (5.73% loss vs. 0.06% increase; Fig. 3) and a significantly greater increase in CBV at the distal femur (1.66% vs. 1.44%; Fig. 3). After adjusting for baseline value and ZA use, leg lean mass and vitamin D levels were both positively associated with end‐of‐study CBV and age at injury was negatively associated with end‐of‐study CBV at the distal femur. CBV increased by 3.0 ± 0.1 mm^3^ for every gram increase in baseline leg lean mass (*p* < 0.001), 440 mm^3^ ± 10 for every ng/mL of baseline 25‐OH vitamin D (*p* = 0.03), and decreased 80 ± 20 mm^3^ for every year of age at injury (*p* = 0.000). After adjusting for baseline value and ZA use, leg lean mass was negatively associated with end‐of‐study CBV at the proximal tibia. CBV decreased by 0.06 ± 0.02 mm^3^ for every gram increase in baseline leg lean mass (*p* = 0.03). These results were similar when excluding women, statin users, and those with acute SCI (injury duration <18 months) and are presented in Table 3. These models explained 98% of the variation in end‐of‐study CBV at the distal femur and 99% of the variation at the proximal tibia.

**Table 3:** Strength of the Zoledronic Acid + Rowing Effect (Rowing‐Only Group as Reference) in Models Adjusting for Baseline Values and Clinical/Demographic Factors

	Cortical bone volume (mm^3^)		Cortical Thickness Index (mm)		Buckling ratio (BR^e^)	
Proximal tibia	β ± SE	*p*	β ± SE	*p*	β ± SE	*p*
Fully adjusted, all participants (*n* = 20)	345 (109)^a^	0.006	0.012 (0.004)	0.013	4.51 (1.73)	0.019
Fully adjusted, chronic SCI only (*n* = 18)	410 (100)^a^	0.001	0.014 (0.005)	0.007	5.20 (1.80)	0.012
Fully adjusted, males only, no statin users (*n* = 16)	404 (91)^a^	0.001	0.017 (0.004)	0.001	5.52 (1.35)^b^	0.001
Distal femur						
Fully adjusted, all participants (*n* = 20)	471 (225)^c^	0.05	0.016 (0.006)^c^	0.009	5.47 (2.04)^d^	0.015
Fully adjusted, chronic SCI only (*n* = 18)	490 (256)^c^	0.076	0.017 (0.006)^c^	0.015	6.20 (2.10)^d^	0.009
Fully adjusted, males only, no statin users (*n* = 16)	400 (256)^c^	0.14	0.014 (0.006)^c^	0.04	4.94 (2.28)^d^	0.05

All models are adjusted for baseline bone geometry values.

SCI = Spinal cord injury.

^a^ Adjusting for baseline leg lean mass (*p* = 0.007 to 0.04).

^b^ Adjusting for total rowing work (*p* = 0.05).

^c^ Adjusting for age injury (*p* = 0.000 to 0.003), baseline leg lean mass (*p* = 0.000 to 0.001), baseline 25‐OH vitamin D level (*p* = 0.01 to 0.03).

^d^ Adjusting for age at injury (*p* = 0.001 to 0.02), baseline leg lean mass (*p* = 0.000 to 0.01).eBR has no units; beta coefficients are multiplied by 1000 here for ease of interpretation.

**Figure 4:**
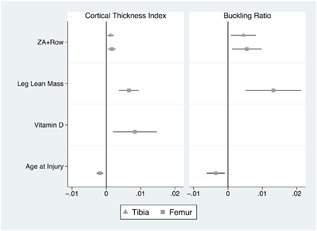
Factors associated with end‐of‐study bone geometry. Confidence intervals presented for cortical thickness index (CTI) and buckling ratio (BR) adjusted for baseline values (rowing‐only group is the reference) at the distal femur or proximal tibia.

**Figure 5:**
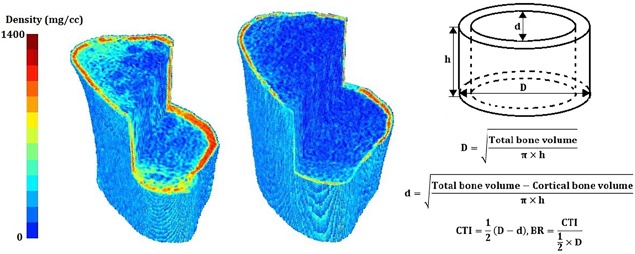
The geometry and density distribution of two tibial metaphysis with high (left; CTI = 0.058, BR = 0.198) and low (right; CTI = 0.021, BR = 0.058) cortical thickness index (CTI) and buckling ratio (BR). CTI and BR are calculated based on the assumption that the region of interest (tibial metaphysis) is a right circular hollow cylinder with outer diameter of D, inner diameter of d, and height of h.

These changes were associated with greater CTI (0.012 ± 0.004 mm, *p* = 0.013 proximal tibia and 0.016 ± 0.006 mm, *p* = 0.009 distal femur) and BR (4.51 ± 1.73, *p* = 0.019 proximal tibia and 5.47 ± 2.04, *p* = 0.015 distal femur) in ZA users compared with those in the rowing‐only arm (Figs. 4 and 5, Table 3). This corresponded to mitigation of 1% to 7% loss of CTI and 2.5% to 8% loss in BR at the proximal tibia and distal femur (Fig. 3). After adjusting for baseline values and ZA use, the distal femur CTI and BR were both positively associated with leg lean mass and negatively associated with age at time of injury. Additionally, baseline 25‐OH vitamin D levels were positively associated with the distal femur CTI. These results (Table 3) were similar when excluding women, statin users, and those with acute SCI (injury duration <18 months). These models explained 97% to 99% of the variation in CTI and BR at the proximal tibia and distal femur.

### Change in bone geometry based on rowing‐alone

A subgroup analysis of the rowing‐only arm (excluding women and statin users) showed that gains in the CTI and BR at the tibial metaphysis varied in a dose‐dependent fashion based on the total amount of exercise performed (*p* = 0.04 to 0.007; Table 4). When comparing the magnitude of the rowing work and the ZA effect on BR at the proximal tibia, approximately 2.533 kWh of work is equivalent to a 1‐time ZA infusion. The other findings were similar when excluding individuals with acute injury (*n* = 2; Table 3), active lipophilic statin users (*n* = 2; Table 3), and women (*n* = 2; Table 3).

**Table 4:** Dose Effect of Total Rowing Work (per Kilowatt‐Hour) on Bone Geometry in Rowing‐Only Arm

	Compressive Strength Index (mg^2^/mm^4^)		Buckling ratio (BR^a^)	
Proximal tibia	β ± SE	*p*	β ± SE	*p*
Fully adjusted, all participants (*n* = 10)	4.11 (2.26)	0.112	0.487 (2.142)	0.826
Fully adjusted, males only, no statin users (*n* = 8)	4.01 (0.90)	0.007	2.442 (0.864)	0.040

All models are adjusted for baseline bone geometry values.

^a^ BR has no units; beta coefficients are multiplied by 1000 here for ease of interpretation.

## Discussion

We conducted a randomized comparative clinical trial to assess the impact of a 1‐year course of FES‐rowing alone versus FES‐rowing + ZA on bone density and bone geometry in paralyzed men and women with chronic SCI. We found that combination therapy (rowing + ZA) mitigated a clinically significant 2.5% to 8% loss in bone geometric properties compared with rowing‐alone. We report a small dose‐dependent effect of total rowing work that was observed at the proximal tibia. There were no serious adverse events associated with participation in this study. In general, the study interventions were well‐tolerated. Nonserious adverse events were common, with almost half of the enrolled participants reporting a study‐related nonserious event. Acute‐phase reaction was common among those receiving ZA infusion, but these symptoms resolved with acetaminophen. Calcium and vitamin D supplementation was well‐tolerated, but was associated with kidney‐stone formation in 4% of the participants. Musculoskeletal pain was the most commonly reported rowing‐related adverse event. These symptoms may have limited the duration or frequency of rowing, but did not appear to be a factor in study retention. There were no fractures reported with electrical stimulation in this study. This is an important consideration given concern in the literature regarding the safety of electrical stimulation of the paralyzed limbs in individuals with advanced osteoporosis as was seen in this cohort.^29^ This suggests that the electrical stimulation paradigm utilized in this study may be appropriate for individuals with chronic SCI. One individual reported ejaculation with electrical stimulation during training sessions. This observation may support future work exploring electrical stimulation of skeletal muscle for sperm retrieval in men with neurogenic anejaculation.^30^

We also identified several clinical and demographic factors that were associated with gains in bone volume or bone geometry, including leg lean mass at the beginning of the trial, vitamin D level at the beginning of the trial, and age at time of injury. In general, greater baseline leg lean mass and 25‐OH vitamin D levels were both associated with greater end‐of‐study values. Older age at injury was associated with lower end‐of‐study values. These findings suggest that correcting vitamin D deficiency prior to initiating a bone intervention in SCI may maximize efficacy. Similarly, lean mass may be a critical determinant of the bone‐regenerative capacity. It is possible that bone regeneration is compromised in cases of sarcopenia or extreme muscle loss. This finding is in agreement with recent reports that muscle contributes to bone growth and repair, likely via satellite cell activity.^31^, ^32^ We report positive associations between baseline lean mass and end‐of‐study distal femur CBV, CTI, and BR. However, we report the opposite, negative association between baseline lean mass and end‐of‐study proximal tibia CBV. It has been reported that bone loss is greater in the distal lower extremity compared with the proximal lower extremity.^33^ That is, bone loss at the calcaneus is far greater than at the hip in motor‐complete SCI. We also report that bone loss is much greater at the proximal tibia than the distal femur. Therefore, this finding may reflect differences in tibia and femur bone metabolism, bone–muscle interactions, or differences in response to therapy. This finding needs to be confirmed in larger studies. Similarly, additional work is needed to clarify the impact of bone–muscle interactions on bone metabolism in response to various interventions, either pharmacological‐ or exercise‐based. Similarly, our findings suggest that bone‐regenerative capacity may be blunted in older individuals, as has been reported in the general population.^34^, ^35^, ^36^

To our knowledge, this is the first study to detail the effects of combining bisphosphonate therapy with a bone‐forming stimulus, such as FES‐rowing, on bone following SCI. ZA is an antiresorptive therapy and FES‐rowing is thought to be an osteogenic stimulus. Therefore, our a priori hypothesis was that gains would be greater in the group receiving combination therapy. Our findings suggest that FES‐rowing work is an osteogenic stimulus that can be quantified in a dose‐dependent fashion. The magnitude of the rowing effect on bone geometry was much smaller than the ZA effect, with one ZA dose being equivalent to roughly 2.53 kWh of rowing work. In the current study, 2.383 kWh was the maximum amount of rowing performed by any participant, with a mean of 0.917 kWh. This suggests that the osteogenic stimulus based on rowing work was not maximized in this study. Increasing the frequency or duration of training sessions may be one approach in future studies; however, the feasibility of maintaining such a rowing schedule is not clear. Once rowing work is stopped, bone resorption would be expected to resume at prior, or perhaps even greater rates. Additionally, the mechanical loading during the rowing cycle may not be optimal to stimulate bone formation in the paralyzed legs. Our trial design did not include quantification of loading forces on the legs during the rowing cycle. However, a recent assessment of the kinematics and kinetics of FES‐rowing suggested that only modest loading to the lower extremity occurs during the rowing cycle.^37^ Future work may focus on approaches to maximize lower extremity loading during the rowing cycle. FES‐rowing also includes a combination of both electrical stimulation and mechanical loading of the legs during push‐off. Electrical stimulation alone is known to be osteogenic and has been studied in SCI.^20^, ^21^ It is possible that the osteogenic effect of FES‐rowing is entirely attributable to the electrical stimulation alone. Subsequent trials will be needed to distinguish the osteogenic effects of push‐off during the rowing cycle from the osteogenic effects of the electrical stimulation.

Despite prior reports in the literature, there remains controversy regarding the efficacy of bisphosphonates in the treatment of disuse osteoporosis. Our study provides evidence that ZA prevents bone loss in chronic SCI at clinical relevant skeletal sites (distal femur and proximal tibia). Our findings are in agreement with prior reports that bisphosphonates mitigate loss after SCI. Seventy mg weekly of alendronate prevented total body and hip bone loss at 1‐year post‐SCI^8^ and in a different study a 2‐year course of daily alendronate (10 mg) mitigated roughly 8% loss of bone density at the distal tibia following SCI.^(11)^ Similarly, 1 dose of ZA mitigated roughly a 6% loss of hip geometric properties at 6 months after administration in 17 subjects with complete acute SCI (<10 weeks postinjury).^38^ The mitigation of bone loss in these reports (6% to 8%) is comparable to the mitigation of bone loss we report in this study. One limitation of these prior studies was the failure to assess bone loss at skeletal sites clinically relevant after SCI: the distal femur and proximal tibia. These two locations account for the majority of osteoporotic fractures in SCI. Published studies assessed bone density or bone geometry at the hip or ankle as surrogate sites for the knee. Our study focused on bone geometry and bone volume at both the distal femur and proximal tibia. Moreover, our primary outcome measure was change in bone geometry at the distal femur and/or proximal tibia, not bone density. Given that only small changes in bone density (1%) by DXA at the hip have been reported in either pharmacological‐ or exercised‐based clinical trials in the general population, we hypothesized that DXA might not be sensitive enough to detect changes in bone based on the study interventions. Furthermore, no literature exists on the detection of therapeutic bone response at the knee. Our original study design included confirmation of the CT findings by DXA at the knee. However, DXA results were only available in a limited number of participants; therefore, this could not be accomplished.

In this study rowing + ZA was associated with clinically meaningful improvements in bone geometric properties at the skeletal sites most frequently fractured after SCI: the distal femur and proximal tibia. Multiple bone geometry indices were calculated in this study based on CT scans. However, our analysis focused on three primary indices: CBV, CTI, and BR. These three indices were selected based on their strong association with prevalent osteoporotic fractures in chronic SCI (manuscript in preparation). Although more work is needed to validate fracture prediction based on bone geometric properties after SCI, it is likely that mitigation of 2.5% to 8% of loss in the CTI and/or the BR may reduce the risk of subsequent osteoporotic fracture.

There are several limitations of the current study to consider. First, we did not include a ZA‐only arm, and participants randomized to the rowing‐only arm did not receive a sham infusion. This is because the primary intention of this study was to compare a novel intervention under two conditions: (1) alone, or (2) in combination with a drug shown to be effective in this population. Nevertheless, this may be considered a study limitation. Second, study retention and training compliance were both low. This is typical of exercise‐intervention studies in general, but even more so with SCI. Unfortunately, not all scans included sufficient length to examine the diaphysis, which we defined as the 20% to 30% length of the tibia and femur (measured in the distal and proximal directions from the knee, respectively). Because the number of scans that included the full diaphysis was so small we were not able to draw any meaningful conclusions about this site based on the present data set. Despite these challenges of conducting clinical trials in SCI, our study is one of the largest exercise‐intervention studies reported in this population. Third, many of the observed changes were small compared with the resolution of the CT images. Nevertheless, they generally exceeded our error values. Furthermore, the magnitude of the changes in mechanically related variables (eg, CTI and BR) were similar, giving us confidence in these measures. Future studies could benefit from higher resolution images to better understand how these interventions affect bone microarchitecture. Although our study may have lacked power to fully characterize the osteogenic effect of FES‐rowing on bone, we detected significant treatment effects in several measures.

Based on our findings, we conclude that combination therapy consisting of rowing + ZA mitigates a clinically significant 2.5% to 8% loss in bone geometric properties of the knee (distal femur and proximal tibia) in chronic SCI. The rowing effect was detectable only at the proximal tibia, was dose‐dependent, and was much smaller in magnitude than the ZA effect. ZA is a feasible therapeutic option to mitigate bone loss in chronic SCI.

## Disclosures

None of the authors have any conflicts of interest.
